# Digestion of Protein in Premature and Term Infants

**DOI:** 10.4172/2161-0509.1000112

**Published:** 2012-04-23

**Authors:** David C Dallas, Mark A Underwood, Angela M. Zivkovic, J. Bruce German

**Affiliations:** 1 Department of Food Science, University of California at Davis, One Shields Avenue, Davis, CA, 95616, USA; 2 Foods for Health Institute, University of California at Davis, One Shields Avenue, Davis, CA, 95616, USA; 3 Department of Pediatrics, University of California Davis, 2315 Stockton Blvd., Sacramento, CA, 95817, USA

**Keywords:** Premature infant, Term infant, Human milk, Proteolysis, Protein, Digestion, Microbiota, Bacteria, Gastrointestinal tract

## Abstract

Premature birth rates and premature infant morbidity remain discouragingly high. Improving nourishment for these infants is the key for accelerating their development and decreasing disease risk. Dietary protein is essential for growth and development of infants. Studies on protein nourishment for premature infants have focused on protein requirements for catch-up growth, nitrogen balance, and digestive protease concentrations and activities. However, little is known about the processes and products of protein digestion in the premature infant. This review briefly summarizes the protein requirements of term and preterm infants, and the protein content of milk from women delivering preterm and at term. An in-depth review is presented of the current knowledge of term and preterm infant dietary protein digestion, including human milk protease and anti-protease concentrations; neonatal intestinal pH, and enzyme activities and concentrations; and protein fermentation by intestinal bacteria. The advantages and disadvantages of incomplete protein digestion as well as factors that increase resistance to proteolysis of particular proteins are discussed. In order to better understand protein digestion in preterm and term infants, future studies should examine protein and peptide fragment products of digestion in saliva, gastric, intestinal and fecal samples, as well as the effects of the gut micro biome on protein degradation. The confluence of new mass spectrometry technology and new bioinformatics programs will now allow thorough identification of the array of peptides produced in the infant as they are digested.

## Introduction

Each year, more than half a million babies (about 1 in 8 deliveries) are born prematurely in the United States [1]. Survival of the smallest premature infants has increased dramatically in recent decades due to major advances in neonatal medical care [2]. This new cohort of survivors is at much higher risk for various morbidities than term infants [3]. Improving nourishment has the potential to decrease premature infant morbidity risk [4]. Protein is an essential component of infant nutrition, as it is required for growth and development of the neonate. Human milk, while ideal for the term infant, is inadequate to meet the protein requirement of premature infants and must be supplemented for adequate growth [5]. Neither milk from mothers of preterm infants nor from mothers of term infants provides enough protein for premature infant adequate growth [5].

At a minimum, protein nutrition for premature infants must provide sufficient essential and non-essential amino acids for the protein synthesis needed for growth and development. The amount of ingested protein required to meet this minimum requirement varies with an infant's ability to break down dietary protein. Nitrogen balance studies determine the amount of protein required to provide an adequate amino acid supply for the premature infant [6], but provide no information on the specific products of digestion nor their functions. Little is known about the products of protein digestion in premature or term infants. For example, lactoferrin (Lf) and secretory immunoglobulin A (sIgA) survive intact to fecal excretion in term and premature infants [7-10], but little is known about other intact proteins and digested protein products, or the implications of the passage of these intact proteins through the intestinal tract.

Studies have determined in the preterm and term infant the concentrations and activities of major gastrointestinal proteases, including pepsin, trypsin, chymotrypsin, carboxypeptidase B and enterokinase. However, digestion is more complicated than the simple sum of the cleavage patterns of well-known proteases. In addition to the effects of cleavage by major proteases, the effects of less abundant proteases, unknown proteases, the digestive environment (pH, transit time, etc.), breast milk-derived proteases and antiproteases, and microbial digestion likely affect the end result of digestion in an individual.

Incomplete breakdown of dietary protein can benefit or harm the infant, depending on the specific molecule remaining intact. Some peptides and proteins left intact may have beneficial bioactivity in the infant [11]. However, some incompletely broken down proteins can elicit an allergic response in the infant and, therefore, have detrimental effects [12,13]. Incomplete protein degradation can also limit amino acid availability for protein synthesis. Incomplete protein digestion and absorption in the upper intestinal tract also results in the availability of protein in the distal colon, where bacterial fermentation of protein— putrefaction—results in the production of potentially harmful molecules, including ammonia, amines, phenols and sulfides [14].

Through recent improvements in mass spectrometry and bioinformatic programs, it is now possible to identify the array of peptides released by digestion in the infant. Peptides can be extracted from digest using conventional solid phase extraction. Extracted peptides can then be introduced into the mass spectrometer via in-line reverse phase chromatography with electrospray ionization. Peptide ions can then be detected with high mass accuracy detectors such as time-of-fight or Orbitrap. Peptide ions can then be automatically selected for fragmentation and the fragment masses will be determined. All spectral data can be imported into proteomic analysis programs for de-novo sequencing of non-specifically cleaved peptides. These programs can then provide lists of the specific peptide sequence with protein of origin information. Now that all of these components are available, the field of nutrition and pediatrics can determine exactly how dietary protein is broken down in the digestive tract.

## Premature Infant Protein Requirements

**Figure 1:**
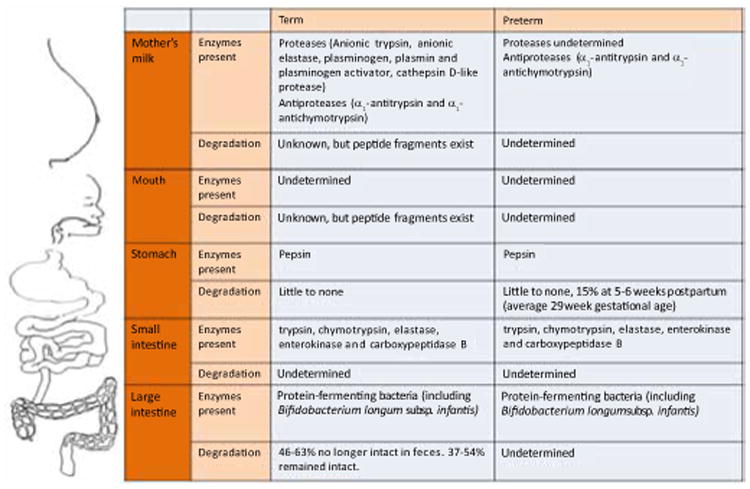
Protein degradation in term and preterm infants.

Protein and amino acid requirements for the human infant, especially the preterm infant, are high because of rapid growth. Premature infants have higher intact protein needs than term infants [15]. For term infants, protein requirements are based on the amount of protein-nitrogen in an adequate intake of breast milk [16]. Researchers have employed two approaches for determining premature infant protein requirement. The first approach sets protein requirements at amounts shown clinically to meet fetal growth rates and nitrogen accretion, while not accumulating potentially harmful protein metabolic products [17]. The second approach sets requirements based on the sum of the amount of protein incorporated into new tissues plus the obligatory nitrogen losses in urine, feces, skin, etc. [16]. Benefits of higher protein intake in premature infants include better growth and protein accretion [18]. Table 1 shows protein requirements for term and preterm infants. For infant formula, an Expert Panel from the American Society for Nutritional Sciences recommended a preterm infant protein requirement of 3.4–4.3 g/kg/d total protein without distinguishing among various stages postpartum [16] (Figure 1).

## Protein Available from Premature Infants Mother's Milk

Milk from mothers delivering preterm is higher in protein than milk from mothers delivering at term (Table 2). Over the first 8 weeks of lactation, the earlier a woman delivers, the higher the protein content of her milk. With time, the milk protein content decreases in both women delivering prematurely and at term [19]. In spite of this higher protein content in preterm mothers, human milk not supplemented with protein is not adequate for the tremendous growth requirements of this unique preterm population [20]. Protein concentration is highly variable among preterm mothers and for a given woman over time [21]. This variability has prompted protocols for individualizing the protein intake of preterm infants [5]. Currently, measuring the protein content of individual samples of milk from mothers of premature infants is technically challenging [22].

## Proteolysis in Milk

A variety of proteases and antiproteases exist in human milk. Ferranti et al. showed via mass spectrometry that over 100 unique protein fragments of β-, κ- and α_s1_-casein exist in human milk from term and premature mothers [23]. Armaforte *et al.* confirmed the presence of low molecular weight casein fragments with 2D-SDS-PAGE and mass spectrometry [24]. This study showed that the casein fragments were present at higher concentrations in premature infants, while the intact caseins were present at lower levels in premature infants than term mother's milk. This data suggests that premature milk undergoes more proteolysis than term milk. Christensen et al. showed that fragments of osteopontin, a common milk protein, also exist in intact term mother's milk [25].

In assessing protein digestion in infants, researchers must consider the effects of proteases and antiproteases secreted in the milk as these enzymes may affect the results of proteolytic degradation at various stages in the gastrointestinal tract.

### Proteases in human milk

Proteases present in human milk include anionic trypsin [26], anionic elastase [26], plasmin (as well as its inactive zymogen precursor, plasminogen, and both tissue-type and urokinase-type plasminogen activators) [27-30], cathepsin D [31-33] and kallikrein [32]. The zymogen of thrombin—prothrombin—was identified in human colostrums, but activated thrombin has not yet been reported in milk [32]. Plasmin cleaves on the C-terminal side of lysine or arginine residues [25]. Cathepsin D, an aspartic endopeptidase, cleaves predominantly between two hydrophobic amino acids, particularly when following leucine [25]. Protease activity in term milk decreases across lactation stages [34,35]. Plasmin activity is higher in premature mother's milk than term milk [24]. Fragments of casein created by plasmin cleavage were identified by Ferranti et al. [23]. Researchers have not yet determined the concentrations and activities of proteases in preterm mother's milk. Proteases in human milk may function to initiate digestion of protein for the infant. The decrease in protease activity in human milk coincides with the increase of the infant's own degradative capacity.

### Antiproteases in human milk

Antiproteases in human milk may function to protect human milk proteins from degradation. The balance of proteases and antiproteases in human milk may be important in guiding protein-specific and time-dependent digestion of proteins within the mammary gland.

Human milk from women delivering at term and preterm contains the antiproteases α_1_-antitrypsin and α_1_-antichymotrypsin from the first day of lactation [35-37]. A_1_-antitrypsin inhibits a wide variety of proteases, including trypsin [38]. A_1_-antitrypsin binds covalently to and irreversibly deactivates trypsin *in vitro* [39]. A_1_-antichymotrypsin inhibits chymotrypsin and chymotrypsin-like serine proteases such as neutrophil cathepsin G and mast cell chymases [40]. A_1_-antitrypsin and α_1_-antichymotrypsin concentrations decline in concentration across lactation from day one to 2 weeks postpartum in both term and preterm milk [35,37]. However, both α_1_-antitrypsin and α_1_- antichymotrypsin are still detectable in both term and preterm milk up to 160 d postpartum with no concentration differences noted between term and preterm samples [35].

Protease inhibitory activity was detected in both term and preterm milk samples from 4–160 d postpartum, and, in some samples, as early as the first day postpartum [35]; however, a comparison of activities in term and premature infants has not been made.

A_1_-antitrypsin has been identified intact in the feces of term breast­fed infants. Therefore, α_1_-antitrypsin can potentially block trypsin and other proteases throughout the term infant digestive tract [7]. Intact survival of other antiproteases such as α_1_-antichymotrypsin in the feces has not been reported.

## Premature Infant Protein Digestion Biology

### Protein degradation in the mouth

Several antiproteases, but no proteases, have been found in adult saliva [41,42]. Whether infant saliva contains proteases or antiproteases and whether breakdown of dietary protein begins in the infant oral cavity is unknown. Small premature infants are fed through a feeding tube bypassing the oral cavity until they are old enough to suck, swallow and breathe in a coordinated fashion. Tube feeding effectively bypasses any oral protein degradation capacity except for that which might occur due to swallowed saliva.

Several studies have reported a select range of milk protein-derived peptide fragments in the saliva of human infants after milk feeding. Term and preterm infant saliva after milk feedings contained peptide fragments derived from milk histatins [43] and proline-rich proteins [44]. Term infant saliva at 3 and 6 months postpartum after a milk feed contained peptide fragments of milk-derived acidic proline-rich phosphoprotein, proline-rich protein 3 precursor and histatin 3 [45]. Problematically, however, these three studies did not determine whether these peptide fragments existed in the intact milk prior to contact with the oral cavity, and thus it remains uncertain whether protein fragment products are due to salivary degradation or due to proteases within the mammary gland.

Secretory IgA appears in term infant saliva as early as the third day of life and increases over time [46]. By 6 months of age, salivary immunoglobulin concentrations are higher in breast-fed infants than formula-fed infants, suggesting that breast feeding stimulates maturation of mucosal immunity and that these proteins are protected from oral digestion [47].

### Protein degradation in the stomach

Chatterton et al. examined the *in vivo* gastric digestion of term infants aged 8 and 28 days at 1 and 3 h after human milk feeding with SDS-PAGE and Western blotting [48]. The study showed that many milk proteins remained intact for at least 1 hr post-ingestion. For example, α-lactablbumin, Lf and secretory component were detected intact after 1 h of digestion in both samples. B-casein was detected after 1 h in the 8 day infant but disappeared in the 1 h sample in day 28. This suggests that digestive capacity increased over time. The 1D SDS-PAGE gel images revealed hydrolysis of milk proteins at 3 h for both 8 and 28 days post-partum. Though Chatterton et al. showed that protein degradation was occurring, they did not determine the sequences of the peptides released from enzymatic cleavage.

Proteolysis in the stomach is highly influenced by pH. The generation of hydrogen ions is mediated by endocrine (via gastrin), neurocrine (via acetylcholine) and paracrine (chiefly via histamine) pathways. Typically, highly acidic pH causes protein denaturation (the loss of secondary and tertiary protein structure), which usually decreases protein resistance to protease cleavage [49,50]. By 14–15 weeks gestation, the structural development of the stomach is complete, including the components for acid production [51]. Both term and preterm infants can produce gastric acid as early as the first day of life [52,53]. Gastric acid production is influenced by parietal cell mass, which increases with growth, and by feeding regimen [54]. Premature infants produce less feeding-stimulated gastric acid than term infants; however, this difference disappears by the end of the first month postpartum [55,56]. This reported finding may not be true of extremely low birth-weight infants as they rarely survived in the cited studies. Due to the low acid production of infants in comparison with adults and because the buffering capacity of term human milk is typically pH 7.0-7.6 [57], neither term nor preterm infants can provide postprandial acid pH in the stomach in the time around birth [58,59]. Premature infants have a gastric pH of 5–7 for up to an hour after feeding, and it drops to pH 3–3.5 at three hours after a feeding [58]. Milk's buffering capacity maintains the postprandial gastric contents at near neutral pH, which is not protein-denaturing.

The extent of gastric proteolysis also depends upon the concentrations and activities of gastric proteases. Pepsin is present in the stomach of fetuses as early as 16 weeks of gestation [60], and is produced at birth by both term and preterm infants. The activity and concentration of pepsin in the gastric fluid of 5–6 week postpartum premature infants (avg. gestational age 29 weeks) prior to introduction of a milk feeding is about five-fold lower than that of adults [61]. Researchers have not yet determined pepsin concentration and activity after mother's milk feeding in term or premature infants. Henschel *et al*. detected a protease in the gastric aspirates of newborn infants within 6–10 h postpartum that was not pepsin [62]. The electrophoretic mobility and immunoreactivity are similar to that of calf chymosin, a protease that cleaves κ-casein and causes casein curdling. This protease is unique in that it disappears from gastric fluid at 10 days postpartum and is not found in adult gastric fluid.

Gastric proteolysis depends upon the activity of the proteases present. Activities of enzymes vary based on pH. Pepsin hydrolyzes proteins optimally at acidic pH [52,54-56,60,62-64] and is denatured when exposed to a pH greater than 7 [56,63]. As postprandial gastric pH is above 5.0 following feedings for at least the first hour [58], pepsin activity is likely to be low early in life. Gastric acid secretion is similar to that of adults by 6 months of age [64], by which time pepsin activity likely becomes significant.

As low gastric pH serves as an antibacterial barrier to the small intestine in adults, higher pH in early infancy may also facilitate bacterial colonization of the infant gut [53].

Mason assayed the gastric contents of 5–13 day old, breast-fed term infants by formol titration and qualitative biuret test methods to determine whether milk proteins were being hydrolyzed in the infant stomach. This study showed that little protein digestion occurred in the stomach of 5–13 day old term infants: no hydrolyzed protein was detected at 90 min post-feeding, and at 180 min, only 1 of 9 samples showed traces of hydrolyzed protein [59]. This may have been due in part to vigorous gastric peristalsis in the second hour post-feeding driving the majority of gastric contents into the duodenum in these infants [59]. Berfenstam et al. detected little or no proteolysis in gastric samples of breast milk-fed term or premature infants (gestational age unspecified) fed human milk over days 6–44 postpartum; however, term and premature infants fed cow's milk did show evidence of gastric proteolysis [65]. Henderson et al. using different methodology, found greater proteolytic degradation in infants fed breast milk than was found in the Berfenstam study. With this method, 5–6 week postpartum premature infants (average 29 weeks gestation) digested 15% of total human milk protein in the stomach [61].

### Protein degradation in the small intestine

In adults, a large portion of dietary protein degradation occurs in the small intestine. Intestinal proteolysis occurs through the combined actions of luminal and brush border enzymes as well as enteric bacterial degradation. As is true for the products of gastric digestion, except for a few specific proteins, the overall products of intestinal digestion of milk in human infants are poorly characterized. This review provides indirect information about intestinal proteolysis via enzyme concentrations and activities in term and premature infants. Additionally, the effect of bacterial fermentation on milk proteins is considered.

#### Luminal proteases

Key luminal proteases in adult intestinal proteolysis include trypsin, chymotrypsin, elastase, enterokinase and carboxypeptidase B. Each of these enzymes is present in both term and premature infants, but typically at concentrations and activities lower than those in adults.

Enterokinase (also called enteropeptidase) is a protease secreted from intestinal epithelial cells in response to food stimulation [66]. Enterokinase is essential for intestinal proteolysis in both adults and infants because it is responsible for the activation of trypsinogen to trypsin [56], which leads to trypsin activation of chymotrypsinogen to chymotrypsin, proteoelastase to elastase and procarboxypeptidase to carboxypeptidase [67]. Two studies showed that enterokinase is present at birth in both term and premature infants, with detection of the enzyme in the duodenal mucosa by 24–26 weeks of gestation [68,69]. Enterokinase is active in both term and preterm infants [70]. Compared with the activity of enterokinase in older children, enterokinase activity was 6% and 20% in 26–30 week gestational age premature infants and term infants, respectively [69].

Trypsin cleaves peptides at the carboxyl side of lysine and arginine [71]. Trypsin concentrations in the duodenum in both preterm and term infants at birth are less than those of adults [72]. During the first week of life, trypsin concentration in the duodenum of premature infants was lower than in term infants, but the concentrations were similar by weeks 2-4 postpartum [72]. By one month postpartum, term and preterm infants' trypsin concentration and activity were similar to those of adults [73].

Chymotrypsin, a luminal pancreatic protease, cleaves on the carboxyl side of tyrosine, tryptophan or phenylalanine [74]. Chymotrypsin concentration in intestinal fluid is similar in term and premature infants at birth and at 30 days postpartum [75], and were 10–60% of adult concentrations [73]. In terms of activity, both at birth and at 30 days postpartum, no difference in chymotrypsin activity was detectable between term and premature infants [75]. Chymotrypsin is present in the feces of both term and preterm infants at birth and does not differ between term and premature infants [76].

Carboxypeptidase B cleaves the basic amino acids arginine and lysine from the carboxy-terminus of peptides and proteins (an excellent complement to trypsin, which produces such substrates) [77]. Carboxypeptidase B is present in similar concentrations and activities in both term and preterm infant duodenal fluids at birth and at 30 days of age. Concentrations and activities were 10–25% of those of 2-year-olds [73].

In summary, in spite of lower enterokinase activity in premature infants, the other major luminal proteases have similar concentrations and activities in term and preterm infants, particularly by 30 days post-partum; however, in the first several weeks of life—the most critical time period in terms of growth, development and survival—preterm infants are likely less capable of digesting proteins.

#### Brush border peptidases

Once proteins reach the brush border of the intestinal lining, a large variety of brush border peptidases such as di- and tri-peptidases begin to further degrade the peptide fragments [78]. Substantial quantities of brush border proteases, including γ-glutamyl-transpeptidase, oligoaminopeptidase, dipeptidylaminopeptidase IV and carboxypeptidase, are present by 22 weeks of gestation [79], and some brush border dipeptidases are present in the fetal gastrointestinal tract as early as 10 weeks gestation [80]. Curiously, γ-glutamyl-transpeptidase concentration was actually higher in the brush borders of 8–22 week gestation fetuses than in adults and children. Concentrations of dipeptidylaminopeptidase IV and carboxypeptidase match adult concentrations as early as 8 weeks gestation in fetuses, and oligoaminopeptidase concentrations in fetuses reach those of adults and children by 22 weeks gestation [79]. Dipeptidylaminopeptidase IV releases N-terminal dipeptides from peptides with penultimate proline, alanine or leucine residues [79]. Aminopeptidase A, however, is not as well developed in infancy—it is far lower in concentration in 8–22 week gestation fetuses than in adults and children [79]. The role of brush border peptidases *in utero* is unclear. Swallowed amniotic fluid has nutritional value to the growing fetus, providing about 15% of protein accretion [81,82]. These enzymes possibly contribute to maximal extraction of amino acids from amniotic fluid. These data suggest that, brush border peptidases are important in nutrition of term and premature infants; however, as no study has determined the activities of these enzymes in term and premature infants, this remains speculative.

Dipeptides and tripeptides can be transported into the intestinal enterocyte [79]. In adults, once small peptides are brought within the enterocyte, these peptides are broken down further to free amino acids [83]. The free amino acids generated are then passed by carrier-mediated mechanisms across the basolateral membrane into the portal blood [83]. Researchers have not yet studied the concentrations and activities of enterocyte internal peptidases in term or premature infants.

#### Bacterial proteases

In addition to the proteases produced by the host, the bacteria of the intestinal microbiota also produce proteases and contribute to the degradation of dietary proteins. A variety of human intestinal bacteria can break down protein, including *Bacteroides* spp., *Propionibacterium* spp. and some members of *Streptococcus, Clostridium, Bacillus* and *Staphylococcus* [84]. Adult intestinal bacteria degrade casein and bovine serum albumin via cellbound and extracellular proteases [85]. These proteins are first broken into peptides and then into volatile fatty acids, ammonia, dicarboxylic acids and various phenolic compounds [85]. The observation that amino acids do not accumulate when these bacteria degrade protein suggests the amino acids are quickly metabolized by the intestinal microbiota. Some bacteria can break down peptides directly, whereas others can only use amino acids that are already free [86]. A wide variety of anaerobes can ferment amino acids, including species from the genera *Peptostreptococcus*, *Campylobacter*, *Acidaminococcus*, *Acidaminobacter*, *Fusobacterium* and *Eubacterium* [87-94]. Some bacteria can utilize both carbohydrates and proteins as an energy source, whereas others are obligate amino acid fermenters [95]. Researchers have not yet determined the amount of bacterial protein degradation in the intestinal tract and colon of term and premature infants. The observation that *Bifdobacterium longum* subsp. *infantis*, a bacterial strain common in the intestinal tract of breast-fed infants, grows on culture media made of pepsin-digested human milk Lf and sIgA suggests that bacterial fermentation of dietary proteins is common in breast-fed infants [96]. A synthesized peptide called prebiotic lactoferrin-derived peptide-I (PRELP-I) that is based on these peptides stimulated growth of *B. infantis* at a concentration of 1–10 μM, but did not stimulate four pathogenic bacterial strains [96]. That observation that Lf and sIgA can survive intact in stools of term and preterm infants [7,10] suggests that such stimulatory peptide fragments could, indeed, survive to support growth of *B. infantis* in the colon, but also that even after exposure to bacteria in the infant large intestine, some milk proteins resist degradation.

### Protein degradation in the colon

A comprehensive comparison of the protein content of ileostomy fluid with that of feces has not been made, so it is not possible to comment further on protein degradation that occurs in the colon. Any proteolysis in the colon would likely be primarily the result of bacterial proteases. Protein-degrading bacteria are present in the colon [14].

## Resistance to Proteolysis

Studies suggest that some milk proteins are particularly resistant to proteolysis in the infant. Such resistance may reflect importance for non-nutritional function.

### Resistance to *in vitro* degradation

*In vitro*, Lf is resistant to digestion, especially at pH approximating that of the infant stomach and when Lf is iron-saturated [97,98]. Likewise, sIgA resists *in vitro* proteolysis by trypsin and pepsin at pH 8.0 and 4.0, respectively [99,100].

### Resistance to gastric degradation

Various milk proteins survive gastric digestion intact in the premature infant, including epidermal growth factor, thyrotropin-releasing hormone, sIgA, immunoglobulin G (IgG) [101-103], Lf and lactalbumin [56]. In the term infant, Lf and sIgA survive gastric digestion intact [7].

### Resistance to complete gastrointestinal tract degradation

A significant fraction of dietary protein remains intact throughout digestion and transit through the gastrointestinal tract. In term infants, the amount of soluble protein excreted in the feces is highest in week 1 (∼1500 mg/24 h) and remains constant (∼700 mg/24 h fecal sample collection) for several months postpartum [7]. The predominant fecal protein is sIgA. In the first 3–4 months postpartum, 10–85% of dietary milk sIgA survived intact in the feces of term infants [7]. Lf was particularly resistant to digestion in term infants, although to a lesser degree than sIgA: 2–6% of Lf remained intact in the first week postpartum and 0.4%–1.6% remained intact for 3–4 months thereafter. Lf continued to be excreted intact in feces for long periods: >10 mg Lf was secreted in a 24-h fecal sample in this study even at 5–6 months postpartum in term infants [7]. However, Lf can be produced within adult human digestive tracts and be excreted intact in stool [104]. Therefore, in order to determine whether fecal Lf represents milk-derived or intestine-derived Lf, stable-isotope labeling of mother's milk is required. Hutchens et al. showed that for two preterm infants, nearly all the urinary Lf was of maternal origin [105]. However, no study has determined the relative amounts of fecal Lf originating from milk compared with the infant intestinal tract via isotope-labeling experiments.

In premature infants fed human milk, the predominant intact fecal proteins are sIgA, Lf and lysozyme (9%, 3% and 0.1%, respectively [10]. Haneberg and Finne detected lysozyme in premature infant fecal samples from 1 to 4 weeks postpartum, with widely variable activity [106]. Although intact lysozyme was assayed in the fecal matter of both premature [10] and term [7] infants, it was detected only in premature infants. Lack of intact lysozyme in term feces suggests either a lesser degree of proteolytic capacity in premature infants or greater production of lysozyme in the preterm mother's mammary tissue. Given the antimicrobial properties of lysozyme, this may represent a protective mechanism.

Lysozyme is produced by the Paneth cells of the small intestine and sIgA is produced by B cells in the lamina propria in the infant. Comparisons between human milk-fed and formula-fed infants suggest that most of the fecal lysozyme and sIgA come from the diet rather than the infant.

## Factors that may Increase Resistance to Proteolytic Degradation of Particular Proteins

The resistance of a particular dietary protein to proteolytic degradation can be influenced by a variety of factors, including phosphorylation, size, charge, tertiary structure, amino acid content and glycosylation [107-111].

Degree of glycosylation can increase a protein's resistance to digestion with trypsin [112] and protease cocktails [113]. The glycosylated forms of human interferon-γ have higher resistance to proteolytic degradation by crude granulocyte protease, purified elastase, cathepsin G and plasmin in comparison deglycosylated forms [114].

Degradation of the glycan component of glycoproteins will affect resistance to proteolysis, and thus the peptide fragments that are produced. However, protein-linked glycan degradation is not discussed in this review.

## Benefits of Incomplete Protein Digestion

Incomplete digestion could be beneficial if biologically active proteins or protein fragments are left intact as a result. Intact proteins or peptide fragments may have biological functions in the intestinal tract or be absorbed and act on other organs [115]. For example, by remaining intact, sIgA can aid in development of the infant immune system and protect against infection [116]. Likewise, Lf, by remaining intact or at least partially intact as lactoferricin, can exert protective antimicrobial actions [117,118].

Studies show a variety of functions for milk protein fragments [119-121]. Milk peptides generated from *in vitro* digests exert an array of biological effects, including behavioral, gastrointestinal, hormonal, immunological, neurological and nutritional responses [11]. For example, casein fragments have a wide variety of *in vitro* and animal model-identified effects, including aiding in calcium absorption (caseinophosphopeptides) [122] and nutrient uptake (casomorphins) [123], improving immune defense (casokinins, casomorphins) [124], acting as antimicrobials (casecidins and isracidin) [125, 126], aiding nerve transmission (casokinins) [11], modulating social behavior, causing analgesia [127], decreasing gastrointestinal transit time [128, 129], decreasing diarrheal effects [130], modulating amino acid transport [131], and stimulating hormonal secretions [132,133]. Researchers have not, however, tested the majority of these effects in term or premature infants.

## Negative Consequences of Incomplete Protein Digestion

Evidence of inadequate protein accretion is common in premature infants, manifesting as poor weight gain, poor length gain, low serum albumin and low blood urea nitrogen levels. Incomplete protein digestion likely adds to inadequate protein intake, resulting in poor protein accretion. Low protein digestive capacity, and thus low capacity for extracting amino acids for protein synthesis, may be part of the reason premature infant dietary protein requirements are higher than those of term infants. Another adverse consequence of incomplete protein digestion is exposure to intact peptides, leading to allergic responses or diarrhea. For example, exposure of some infants to intact cow's milk proteins can cause allergic response that disappears when protein is first hydrolyzed before feeding [12,13]. Incomplete digestion could result in decreased release of biologically active peptide fragments. Finally, the incomplete digestion of proteins in the small intestine results in the passage of these proteins into the distal colon, where bacterial fermentation takes place [84], and this bacterial fermentation results in the formation of potentially toxic metabolites such as ammonia, amines, N-nitroso compounds, phenols , and sulfides [14,134]. However, the long-term effects of exposure to these metabolites on the gut mucosa and epithelial cells remain unclear [14].

## Conclusion and Future Research Directions

Given the high number of premature births and high morbidity in a premature infant population, there is a great need for better understanding of protein digestion and improved dietary approaches. Protein supply to premature infants must not only meet overall protein requirements, but also be provided in forms that allow the release of beneficial biologically active peptides.

In order to better understand dietary protein digestion in infants, researchers now have access to remarkable new tools to carry out proteomic and peptidomic studies on the intact milk, saliva, gastric fluid, urine and feces of both term and premature infants. Proteomic studies will determine what proteins remain relatively intact at each stage of digestion as well as the concentrations of proteases, while peptidomics will reveal the peptide fragments produced from digestion at each point. Fecal transcriptomic studies can also provide information on the amounts of digestive enzymes produced in the term and premature infant gut. Use of proteomics and transcriptomics to identify enzymes may lead to discovery of enzymes important for proteolytic digestion that have not been previously considered or detected. Bacterial sequencing of fecal samples is now possible to determine which protein-degrading species exist in gastrointestinal tracts of term and premature infants. These data will provide insight to the interaction between microbial metabolism and proteolytic breakdown. The development of complex computer models [135] and *in vitro* models of digestion [136] will further add to a more complete understanding of human milk protein digestion in term and premature infants.

## Figures and Tables

**Table 1:** Protein requirements in term and preterm infants.

Gestational age at birth	Preterm (26 weeks gestational age, 900 g average weight)	Preterm (30 weeks gestational age, 1,500 g average weight)	Term
Enteral protein requirement (g/ kg/d)	4 [15]	3.6 [15]	1.98 for first month of life, 1.18 for 4–12 mo. [138]

**Table 2:** Average protein concentration of milks from term and premature mothers over the first eight weeks of lactation (data from [19]).

	Extremely preterm human milk (<28 weeks)	Severely preterm human milk (28–31 weeks)	Moderately preterm human milk (32–33 weeks)	Term human milk
**Average protein concentration (g/dL) for weeks 1–8 of lactation**	2.3 ± 0.5	2.1 ± 0.3	1.9 ± 0.3	1.6 ± 0.4

## Abbreviations

Lf: Lactoferrin
sIgA: Secretory immunoglobulin A
IgG: Immunoglobulin G
